# Comparison of the Accuracy of Two Transfer Caps in Positional Transmission of Palatal Temporary Anchorage Devices: An In Vitro Study

**DOI:** 10.3390/dj11020051

**Published:** 2023-02-13

**Authors:** Vincenzo Quinzi, Simone Ettore Salvati, Valeria Brutto, Giorgia Tasciotti, Giuseppe Marzo, Gianmaria Fabrizio Ferrazzano

**Affiliations:** 1 Department of Life, Health and Environmental Sciences, Postgraduate School of Orthodontics, University of L’Aquila, 67100 L’Aquila, Italy; 2 UNESCO Chair in Health Education and Sustainable Development: Oral Health in Paediatric Age, University of Naples, Federico II, 80138 Naples, Italy

**Keywords:** imaging, three-dimensional, bone screws, orthodontic anchorage procedures, orthodontic appliances, dentistry, operative

## Abstract

The aim of this study was to compare the positional information transfer accuracy of palatal temporary anchorage devices (TADs) of two different brands of transfer caps: PSM and Leone. Thirty plaster casts of maxillary dental arches were chosen for master models. A couple of Leone TADs were inserted in each master model. For each master model, two analysis models were created: using two transfer caps, Leone and PSM, the impressions were taken, the analogues were connected on the transfer caps, and the casts were poured. Using digital methods and equipment, such as a 3D scanner, a 3D analysis and a comparison of the accuracy of the two transfer caps in transferring the positional information of the TADs was then made. The data obtained were anal*yz*ed using the Mann–Whitney U-test at a significance level of α = 0.05. PSM transfer caps showed higher error frequency in almost all measurements. Only two measurements had a larger error in the analysis models made with Leone transfer caps. The Mann–Whitney U-test found a significant difference between the error levels of TADs found in the analysis models created with PSM transfer caps. Leone transfer caps showed greater reliability in TADs positional information transmission.

## 1. Introduction

The need to precisely transfer any type of intraoral information to laboratory technicians has always been the most important aspect in every branch of dental clinical practices, and as such, it has been pushing the research ahead in these fields [1]. Customized impression trays, optimized transfers of specific sizes and types of insertion, dental impression materials of increasingly high performance, and the use of high precision digital technologies [2] are aimed all with reproducing intraoral characteristics in the laboratory in the most accurate and precise way. This also applies to the positioning of palatal temporary anchorage devices (TADs) for which precision between planning and actual positioning is crucial to produce stable and effective skeletally anchored orthodontic devices [3,4].

The use of palatal TADs is gaining ground in orthodontics. Adding a skeletal support to a dental anchorage or creating a fully skeletal one is often needed to carry out expansion in adult patients, distalizations, or other movements, which can be too complex to be managed exclusively with dental anchorages [5,6,7]. Digital technologies provide high accuracy in transmitting positional information, besides providing a simplification of elaborative procedures. The newest methods include the creation of templates from the analysis of cone-beam computed tomography (CBCT) and it’s matching with the intraoral scan: after choosing the position of TADs on a computer-aided design (CAD) project, a template for TADs insertion is created and then used as a guide during the clinical session. The result is a greater accuracy in reproducing the insertion information and a reduction in operational steps [8,9]. Despite these advantages, palatal TADs free-hand positioning and subsequent transfer of their position is still very popular and widely used [10].

If TADs are placed free-hand, according to precise criteria regarding the palatal anatomy [11], it will be necessary to accurately transfer positional information to the dental technician. It is essential that the device can be fitted passively without being affected by directional forces or the stability of the TADs. To achieve this goal, analog transfer caps are usually applied on TADs and then embedded in dental impressions [12]; another way includes the use of scan bodies capable of being detected by intraoral scanners [13]. As it is widely known from different studies performed in implant dentistry, these two positional information transfer techniques provide a similar level of precision [14,15,16]. This, together with the lower economic investment required, are probably the reasons why many clinicians still turn to the analogue method for transferring positional information to the laboratory.

In the orthodontic field, several manufacturers propose their own systems for manufacturing TADs. It sometimes happens that some components of kits from different manufacturers are interchangeable. This came in particularly handy during the first two years of the recent SARS-CoV-2 pandemic period due to a general shortage of materials from various suppliers [17]. This shortage also affected the dental sector [18]. In that period, many orthodontists and orthodontic technicians, as well as for professionals working in our university’s dental clinic, sought different solutions to be able to continue producing skeletally anchored appliances. We have, therefore, noticed the interchangeability offered from the analog transfer caps produced by two manufacturers: PSM (PSM Medical Solutions, Gunningen, Germany), and Leone (Leone SpA, Sesto Fiorentino, Florence, Italy). They are indeed interchangeable when used with Leone-brand TADs for expanders and their laboratory analogues.

The aim of our study was to see whether this interchangeability could lead to a decrease in transfer accuracy, and investigate which of the above-mentioned two analog transfer caps is more accurate for transferring Leone TADs positional information. Through digital processes, anal*yz*ing data obtained using scan bodies to avoid any interference and positional changes, the authors have anal*yz*ed the positions of TADs in the final models in order to obtain information about the predictability of these systems. The null hypothesis was that both PSM and Leone transfer caps had the same level of accuracy in TADs positional information transmission.

## 2. Materials and Methods

The entire study was performed using materials that are normally used in dental offices to simulate the conditions of daily clinical practice.

### 2.1. Analog Stage

The planning of a skeletal anchorage for maxillary expansion was simulated. Thirty plaster casts of maxillary dental arch were selected from the patient archive of the university dental clinic. The inclusion criterion was good identification of palatal anatomy on the models. The thirty selected models have been catalogued as master models and referred to as codes from 1M00 to 1M029.

Two Leone TADs of 2 mm diameter and 9 mm length were inserted in each master model. The TADs have been placed in the anterior paramedian region, at a 4–5 mm distance from the palatal midline, between the second and third palatal rugae. This is usually considered the most appropriate anatomical position for this type of procedures [4]. A pilot hole was prepared in the chosen position before of the TADs insertion. The pilot hole had 1.5 mm diameter and 9 mm length, long enough to host the TADs and narrow enough to allow stable insertion. Insertion of the TADs was made permanent by applying cyanoacrylate glue to the pilot holes before placement. 

From each master model, two analysis models were reproduced: one using the Leone transfer cap and, the other using the PSM transfer cap. The Leone transfer caps were positioned and fixed, using the appropriate screwdriver tool, on the TADs’ heads on the master models, then a polyvinyl siloxane impression using Elite HD Putty and Light (ZHERMACK GmbH, Marl am Dümmer, Germany) with double-phase two-component technique was taken. A plastic impression tray was used for the impression. The impression tray was previously modified by making a window at the TADs on the palate of the master model so that the transfer caps screwed onto the TADs could be accommodated. After hardening of the impression material, the Leone transfer caps were unscrewed from the TADs and the impression was then removed from the master models. The Leone transfer caps remained included in the impression. A couple of the Leone laboratory analogues for each impression were then connected and screwed onto the Leone transfer caps, and the impressions thus composed were used to realize the first thirty analysis models. A type 4 extra hard resin reinforced dental plaster was used to process this last step. A similar process was made using the PSM transfer caps to obtain the other thirty analysis models. The PSM transfer caps, due to their clip-on mechanism, remained directly embedded in the impression material without needing to modify the impression tray or unscrew the caps before removing of the impression. This was simply necessary when inserting the Leone laboratory analogues into the impression at the PSM transfer coping holes in order to be ready to realize the second cast. At the end of the whole process, to distinguish the two sets of analysis models, the letters L and P were added before their respective names thus naming from L-1M00 to L-1M29 for the analysis models made with Leone transfer caps and from P-1M00 to P-1M29 for the analysis models made with PSM transfer caps.

**Figure 1:**
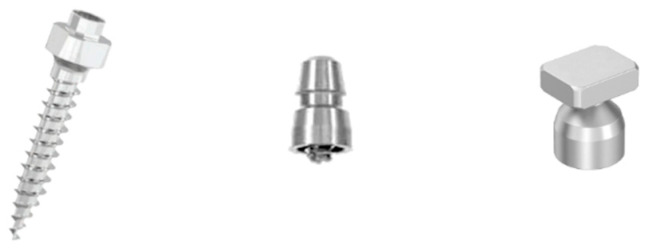
The Leone TADs and the two transfer caps, Leone and PSM, used in the study.

The Leone TADs and the two transfer caps, Leone and PSM, used in this study are shown in Figure 1.

### 2.2. Digital stage

**Figure 2:**
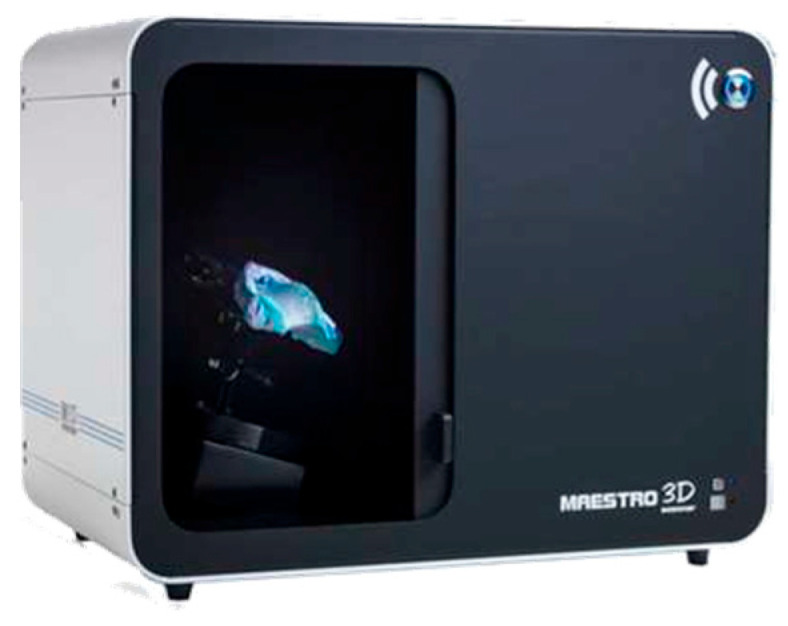
The benchtop scanner used for scanning models: MDS-500 (AGE Solution S.r.l., Pisa, Italy).

**Figure 3:**
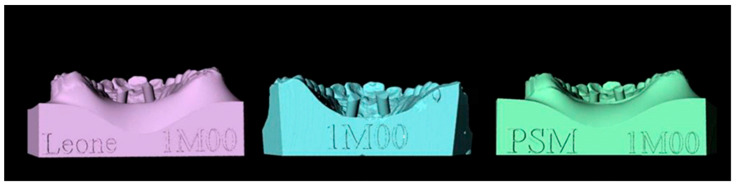
Models processed with Maestro 3D Dental Studio: in the center the master model, on the left the analysis model made with Leone transfer, on the right the analysis model made with PSM transfer.

**Figure 4:**
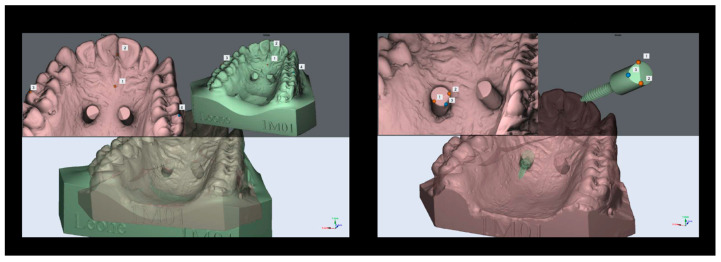
The left image shows the matching between models, the right shows the matching between the TAD and the scan body.

At the end of the preparation process described above, the thirty master models and their respective analysis models containing the laboratory analogues were sent to the 3D Leone department (Sesto Fiorentino, Firenze, Italy) where the following steps took place:

1. Digitization of the models
  - Leone scan bodies for TADs were screwed onto the emerging head of the TADs and TAD analogues.
  - Models were digitally scanned using a MDS-500 dental scanner and MAESTRO 3D dental scan software (AGE Solution S.r.l., Pisa, Italy) (Figure 2 and Figure 3).
  - Each digital master model was superimposed with its respective digital analysis models using some teeth and palatine rugae as reference points (Figure 4). In this way, a single reference system for each set of models was defined.
2. Geometry alignment
  - From the Leone digital library, stereolithography files (STLs) of the scan body and TAD were selected and then coupled together using MAESTRO 3D software (version 5).
  - A coupled scan body and TAD unit were matched with each scan body of every set of models previously embedded as single reference systems using MAESTRO 3D software (Figure 4).
3. Setting the measurement method

**Figure 5:**
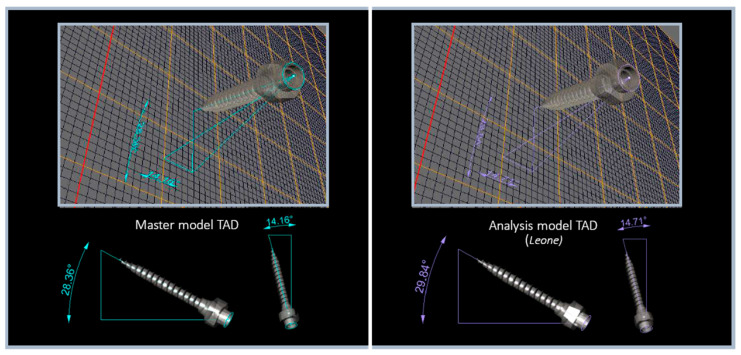
Starting from the axis of the TADs in the master models (in the **left image**), the degrees of rotation and deviation from the *x* and *y* axes of the TADs in the analysis models (in the **right image**) were defined.

To define the position of the TADs in the area, their axis have been outlined and the angle between their axis and the reference planes were measured and compared (Figure 5). The identification of the center of the TADs’ heads on the master models was used to define the displacement of the analysis models TADs. Rhinoceros 4 software (Robert McNeel & Associates, Seattle, WA, USA) was used for the whole process and measurements.

4. Measurements

**Figure 6:**
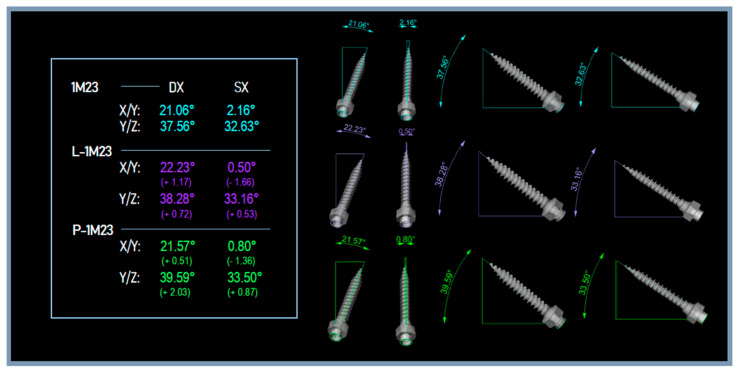
The angles of the TADs of the master model 1M23 and of the related analysis models (L-1M23, P-1M23).

**Figure 7:**
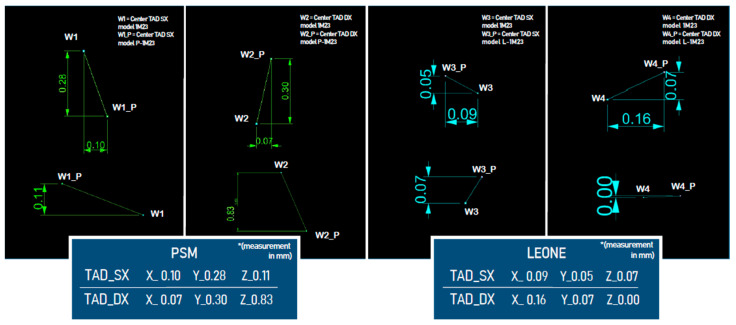
The distance of the centers of the TADs’ heads of the master model 1M23 and of the related analysis models (L-1M23, P-1M23).

**Figure 8:**
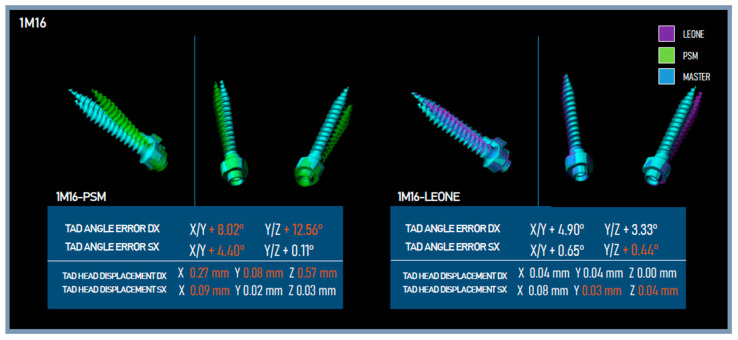
TADs error levels for the analysis models L-1M16, P-1M16.

For all TADs of the master models and their analysis models, the angles were measured and the difference between the measurements was calculated (Figure 6). The displacements of the centers of the TADs’ heads of the analysis models and of the master models were measured (Figure 7). Deviation between the STLs of the TADs and the levels of error in terms of angles and displacements were revealed (Figure 8).

### 2.3. Data Collection

The errors, in terms of angles and displacements, that occurred on different planes and in different spatial directions were listed as Angle*_xy*, Angle*_yz*, Disp*_x*, Disp*_y*, Disp*_z*. To differentiate the parameters of the right and left TADs, *dx* and *sx* indications, respectively, have been added to the previously listed terms. The values for the error parameters described above were collected for all sixty analysis models (thirty for those made with Leone transfer caps and thirty for those made with PSM transfer caps).

### 2.4. Statistical Analysis

The error values of the two transfer caps have all been reported as positive values because the point was to record the absolute deviation of each TAD from its reference sample. We wanted to compare error values produced by the transfer caps of the two different manufacturers, therefore the data were statistically analysed using Mann–Whitney U-test at a significance level of α = 0.05. The statistical analysis was performed using IBM SPSS Statistics software, version 28.0 (IBM Corp, Armonk, NY, USA).

## 3. Results

**Figure 9:**
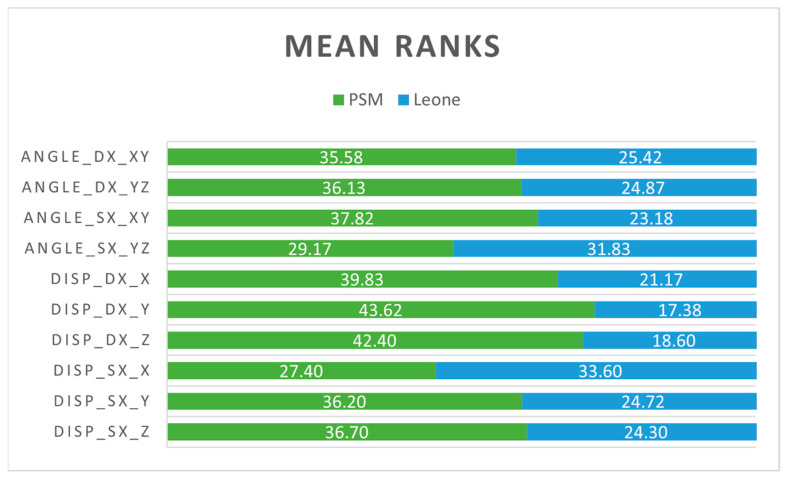
The mean ranks related to the error levels produced by the transfer caps in terms of angles and displacements in the different planes and spatial directions.

The mean ranks related to the error levels produced by the two transfer caps in terms of angles and displacements that have occurred on different planes and in different spatial directions are shown in Figure 9. The absolute frequency of error is greater in the analysis models made with PSM transfer caps for almost all measurements, except for the angle on the *yz*-plane and the deviation in the *x*-axis direction regarding the left TADs. For the latter two measurements, the frequency of error is greater in the analysis models made with Leone transfer caps, with 31.83 in Angle_sx_*yz* and 33.60 in Disp_sx*_x.*

According to the data shown in Table 1, the Mann–Whitney U-test found a significant difference between error levels of the TADs in the two sets of analysis model for almost all measurements, except for the angle on the *yz*-plane and the deviation in the *x*-axis direction regarding the left TADs. For the latter two measurements, the Mann–Whitney U-test did not find significant differences between error levels of the TADs in the two set of analysis model.

## 4. Discussion

As previously shown in the results, statistical significance exists for measurements whose frequency of error is greater in the analysis models made with PSM transfer caps than for measurements whose frequency of error is greater in the analysis models realized with Leone transfer caps. This suggests that PSM transfer caps have a lower level of accuracy in transferring TAD position information than Leone transfer caps. This could lead to a few problems, mainly related to the difficulty of coupling the appliance during placement in the patient’s mouth, and to potential instability of the appliance in the long term [19,20].

The authors performed the experiment in such a way as to reduce the risk of introducing inaccuracies and errors of various kinds as much as possible. The manufacturer’s directions were strictly followed for all materials used. In addition, impressions were taken with the double-phase two-component technique which, as described in the literature [10], yields optimal results in terms of accuracy. As demonstrated by Iodice et al. [8], the positioning of TADs by the direct method is safe and accurate when performed in the anterior region of the palate. It is therefore reasonable to assume that it is the different characteristics possessed by different transfer caps that determine the greater or lower accuracy in the transfer of positional information.

Regarding these insights, given the paucity of studies in the orthodontic field, we are forced to examine the implantology literature field. Several studies have attempted to compare positional information transfers performed using transfer caps with a screw attachment method versus those with a clip-on mechanism [21,22,23]. They all concluded that the clip-on mechanism is less accurate than the screw attachment method. Some factors that can play a major role in the loss of accuracy of the clip-on mechanism are the tactile sensation and the snap mechanism that indicate proper seating of transfer cap. In some cases, the dentist feels no snap and improperly assumes that the transfer cap is properly seated [24]. Furthermore, the connection of the analog to the transfer cap with the clip-on mechanism may result in movement of the cap into the impression material. Certainly, the stability and repeatability of coupling achievable using transfer caps with a screw attachment method can ensure greater accuracy in transferring the positional information of TADs. From a clinical point of view, however, the lower level of accuracy in transferring TAD position information demonstrated by the PSM transfer cap may not be significant. Fitting defects could easily be resolved in the case of appliances with a less rigid structure, incorporating fewer tooth elements, or for those intended for less complex orthodontic movements [25,26]. However, these are assumptions that cannot be clinically verified in an in vitro study.

In the context of our experiment, we should point out that PSM transfer caps showed some difficulties during coupling to the TADs on some of the master models because of the size of the head of the transfer caps. This problem has been highlighted when PSM transfer caps were used in models with a contracted palate, where a transfer cap on one side bumps against the contralateral, not allowing a complete insertion to be achieved. It is important to specify that this problem is dependent on the head size of the PSM’s transfer caps and not on the coupling with Leone’s TADs, as it can also occur during coupling with the PSM’s TADs.

### Study Limitations

The use of a plaster model for making master models may have posed a risk for the potential instability of Leone TADs following their insertion and during subsequent phases of the study. However, this option represented the easiest way to perform an in vitro study.

## 5. Conclusions

The Leone transfer caps, which are less bulky and are screwed, guarantee a greater accuracy in the TADs positional information transmission even if their management is slightly more difficult due to the need of modifying the tray before taking the impression and of unscrewing to allow the impression removal. 

The PSM transfer caps, thanks to their clip-on mechanism, do not require any special maneuvers when taking the impression, but due to an imperfect fit, they can influence the accuracy in the positional information transmission of the TADs. Furthermore, due to their larger rectangular head, in those clinical conditions where there is a significant maxilla transverse contraction, they may bump each other, and thus their insertion could be more difficult, making them even less secure.

## Figures and Tables

**Table 1:** Statistical data of the Mann–Whitney U-test performed on grouping variables.

	Angle_dx *xy*	Angle_dx *yz*	Angle_sx *xy*	Angle_sx *yz*	Disp_dx *x*	Disp_dx *y*	Disp_dx *z*	Disp_sx *x*	Disp_sx *y*	Disp_sx *z*
Mann–Whitney U	602.500	619.000	669.500	410.000	730.000	843.500	807.000	357.000	623.500	636.000
Standard Error	67.638	67.638	67.637	67.639	67.615	67.611	66.341	67.516	67.571	67.580
Standardized Test Statistic	2.255	2.499	3.245	−0.591	4.141	5.820	5.381	−1.377	2.568	2.752
*p*	0.024 *	0.012 *	0.001 *	0.554	<0.001 *	<0.001 *	<0.001 *	0.168	0.010 *	0.006 *

Angle_dx/sx *xy*/*yz*, angle of error on the *xy*/*yz*-planes of the right/left TADs; Disp_dx/sx *x*/*y*/*z*, displacement error in *x*,*y*,*z* spatial directions of the right/left TADs. * *p* < 0.05.

## Data Availability

Data are available from the corresponding Author upon request.
